# Real-world evaluation of an automated EEG spike detection software in a tertiary centre compared to a clinical reference standard

**DOI:** 10.1007/s00415-026-13636-0

**Published:** 2026-01-30

**Authors:** C. Cook, A. Auwal, S. Eglese, B. Hywel, M. A. Ellul, B. D. Michael

**Affiliations:** 1 The Walton Centre NHS Foundation Trust, Liverpool, UK; 2 The Liverpool Interdisciplinary Neuroscience Centre, Liverpool, UK; 3 The University of Liverpool, Liverpool, UK; 4 Liverpool Interdisciplinary Neuroscience Centre, 2Nd Floor Clinical Sciences Centre, Aintree University Hospital, Lower Lane, Fazakerley, Liverpool, L9 7AL UK

**Keywords:** Electroencephalography, Machine learning, Interictal epileptiform discharge, Artificial intelligence

## Abstract

**Background:**

Interictal epileptiform discharges (IEDs) are transient spikes or waves that occur in electroencephalography (EEG) records and can help support the diagnosis and classification of epilepsy. High-throughput machine learning models aim to automate the detection of IEDs. Previous evaluations of machine learning models have reported non-inferiority compared to human experts, but these studies predominantly use small datasets of pre-selected, ‘IED rich’ records, which are not representative of clinical practice. Therefore, this study aims to analyse the accuracy of machine learning models in a large, routine, clinically representative cohort.

**Methods:**

All routine EEGs performed in a large regional hospital in England were identified between June 2024 and February 2025. EEG records were run through the commercial machine learning model P15 and automated IED reports generated. The sensitivity, specificity, positive and negative predictive value of P15-detected IEDs were evaluated using the final clinical report as a reference standard.

**Results:**

Of 484 EEG records, 53 were reported to contain at least one IED in the final clinical report. At P15’s default sensitivity setting, sensitivity for IED detection was 81.1% (95% CI:77.6–84.6), specificity 59.9% (95% CI: 55.5–64.2), positive predictive value 19.9% (95% CI:16.3–23.5) and negative predictive value 96.3% (95% CI:94.6–98.0).

**Discussion:**

This large-scale study of a machine learning model for identification of IEDs in a representative clinical population found a high negative predictive value suggesting that this may be a useful tool to rule out IEDs. However, the low positive predictive value demonstrates the potential for over-calling IEDs in routine EEGs. Future research should evaluate machine learning models alongside clinical feedback before this approach can have sufficient utility in direct clinical care.

## Introduction

Interictal epileptiform discharges (IEDs) are abnormal electrical patterns in the brain that happen between seizures and occur transiently in electroencephalography (EEG) recordings. IEDs can occur as spikes or sharp waves and can arise from different areas of the brain. Their presence can help support a diagnosis of epilepsy and its classification [1]. Identification of IEDs is conventionally performed by a neurophysiologist based on visual inspection. EEGs can be days long, meaning that manual detection of IEDs is resource intensive, often requiring hours of intense focus by a physiologist [2]. Consequently, in non-specialist settings, reporting of EEGs can be delayed or unavailable. Additionally, due to the transient nature of IEDs, there is often a high degree of interrater variability [3]. Automated IED detection is therefore a long-term goal; aiming to reduce workloads, standardise IED detection, and expedite reporting.

Persyst is a commercially available machine learning based software that aims to automatically detect IEDs. The accuracy of Persyst in IED detection has previously been evaluated and is summarised in Table 1. These previous studies use various datasets, reference standards and express the accuracy of Persyst in different ways.

**Table 1:** Summary of previous evaluations of Persyst's accuracy

Study	Aim	Dataset	Reference standard	Comparison measures	Results	Limitations
Mubeen., et al., (2024)	Compare Persyst 14 with Encevis (another spike detection model) and human experts for detection of individual IEDs	125 EEG recordings of patients with diagnosed genetic epilepsy	Consensus agreement of (2/3) epileptologists. ‘Missed’ IEDs that were detected by either model then evaluated by the 3 epileptologists and integrated into the reference standard if found to be relevant	Sensitivity, precision, specificity, F1 score	Persyst sensitivity 67%, highly specific for non-IED detection windows, precision 0.49	Testing dataset:
						Split into epochs
						Preselected EEGs (124/125 were abnormal)
						Study population homogenous
						Reference standard:
						Questionable validity of including IEDs detected by Persyst/Encevis into reference standard
						Uses consensus agreement of very few raters
						Analysis:
						EEG not analysed on whole level
						No analysis of interrater agreement
Reus et al*.,* (2022)	Comparison of Persyst 14, Encevis and BESA (another spike detection model) at categorisation of EEG epochs into IED containing or not	30 prolonged EEGs from epilepsy monitoring unit. Representative 30 min epochs extracted from these and then further split into 10 s epochs	Consensus agreement between 3 experts for presence or absence of IEDs in an epoch	Kappa scores, sensitivity and specificity	In 30 min epochs, Persyst had 95% sensitivity and 88% specificity for detecting the presence of IEDs. In 10 s epochs, sensitivity was 82% and specificity was 96–99%. Interrater agreement (kappa) between Encevis, Persyst and human experts was similar to the agreement between experts and original clinical reports	Testing dataset:
						Split into epochs
						Preselected EEGs
						Homogenous study population
						Reference standard:
						Consensus agreement of only 3 experts
Kural et al*.,* (2022)	Comparison of three algorithms (including Persyst 14) at IED detection and also comparison of algorithm + human in the hybrid approach	Sixty 20 min EEG recordings containing sharp transients (epileptic or not) from 30 patients with epilepsy and 30 patients who had paroxysmal non-epileptic events. All had to have had video-EEG and associated diagnosis from this (epilepsy or not). For patients with epilepsy, the IED marked in the routine EEG had to be concordant with the ictal event recorded on video-EEG	Epilepsy or not, based on diagnosis by video-EEG	Sensitivity, specificity	Alone, Persyst was 3.33% specific but 100% sensitive for detecting epilepsy. Hybrid approach: specificity 96.67% and sensitivity 76.67%. The overall accuracy of Persyst was statistically non-inferior to complete human review, but with a significantly reduced review time	Testing dataset:
						Strict inclusion criteria –may not be representative of the general population and the IEDs may be of different morphologies. Focal epilepsy may be overrepresented in EMU setting
						Preselected EEGs
						Reference standard:
						Robust reference standard, but possible that some sharp transients (in the non-epileptic group) that were labelled as other benign activity could have been IEDs (due to original misdiagnosis of epilepsy or occurrence of IEDs in non-epileptics)
Duong et al*.,* (2021)	Test accuracy of a spike detection model (Resnet). Model subsequently compared with Persyst	For testing Persyst, 8 EEGs with generalised IEDs and 11 normal recordings randomly selected from two sites	Clinically reported EEGs	Sensitivity and specificity (at EEG level). Sensitivity and precision (PPV) (for individual IEDs)	100% sensitive and 58% specific for detecting abnormal EEGs. For individual IED detection it was 82.7% sensitive and the precision (PPV) was 57%	Testing dataset:
						Preselected EEGs
						Very small testing dataset (n = 19)
						Reference standard:
						Clinically reported – questionable accuracy
						Analysis:
						Study not looking specifically at Persyst
Halford et al*.,* (2018)	Evaluate the performance of academic and private practice neurologists in the detection of IEDs in routine EEG recordings. Studies also run through Persyst at multiple sensitivity settings	200 partially randomly selected epochs of 30 s. 50 were normal, 50 contained IEDs, 50 contained ‘difficult to interpret IEDs’ and 50 contained other benign paroxysmal activity	19 academic and 16 private practice neurophysiologists	Pairwise sensitivities and false positive rates	At all sensitivity settings, Persyst had a higher false positive rate than all EEG raters	Testing dataset:
						Preselected EEGs
						EEGs split into epochs
						Analysis:
						Study not looking specifically at Persyst
						Does not report actual values for Persyst’s performance
Scheuer et al*.,* (2017)	Comparison of Persyst with 3 neurophysiologists	40 prolonged EEGs preselected as containing IEDs (n = 35) with normal distractors (n = 5)	3 neurophysiologists	Pairwise sensitivities and false positive rates	At a sensitivity setting of 0.1 (non-default setting), Persyst had a sensitivity of 43.9% and a false positive rate of 1.65/min. This is reportedly non-inferior to experts. 68% sensitivity for consensus spikes (spikes marked by 3 neurophysiologists)	Testing dataset:
						Preselected EEGs
						Only 40 EEGs used (although prolonged)
						Homogenous cohort (EEGs all performed at single site)
						Reference standard:
						Only 3 raters used
						Analysis:
						Accuracy measured for detection of individual IEDs only. Not mentioned whether Persyst successfully identified the normal records
						Raters had the same training, from the same institute
Jing et al*., (2019)*	Development of IED detection model, SpikeNet. Persyst was used as a comparison	1051 EEGs which were partitioned into training, validation and test sets	Consensus agreement between 8 neurologists: a definite IED was where > 6 neurologists annotated, definite non-IED was < 2	AUC for individual classification of IEDs	Persyst had an AUC of 0.882	Testing dataset:
						Size of the testing dataset unclear
						Reference standard:
						Consensus agreement
						Analysis:
						Study not focussing on Persyst
						Sensitivity and specificity not reported
Tjepkema-Cloostermans et al*.,* (2024)	Testing of SpikeNet on clinical routine EEGs. Compared with Persyst	22 EEGs with spikes and 28 as controls	Consensus agreement between 7 expert raters	Sensitivity and specificity	Persyst had 64.6% sensitivity and 98% specificity for the detection of individual IEDs. Non-inferior to the human experts	Testing dataset:
						Small dataset
						Split into epochs
						Reference standard:
						Consensus agreement favours visually obvious IEDs
						Analysis:
						Study not focussing on Persyst
						Reports on individual IEDs only

Notably, some evaluations claim non-inferiority of Persyst compared to humans. First, based on pairwise sensitivities and specificities being in the range of three human experts, it has been claimed that Persyst passes a ‘statistical Turing test’ in individual IED detection [4]. Secondly, it has been shown that, when used in a hybrid approach of Persyst supervised by a human expert, it performed as well as conventional human review while also significantly reducing review time [5]. These findings are not congruent with anecdotal and qualitative evidence reviewing Persyst’s use clinically; a recent qualitative analysis identified low clinician trust in the software [6], and automated IED detection models are scarcely used clinically [7]. Low clinician trust, despite reported human-level accuracy, may be due to limitations associated with previous studies. Persyst has not been evaluated in a large clinical dataset of routine EEGs.

A key issue in previous evaluations of Persyst’s accuracy is the selection of an appropriate reference standard, because there is a high degree of interrater variability in IED detection [3]. Some studies use consensus agreements, which help eliminate interrater variability but can favour more visually obvious IEDs [8–11]. To avoid consensus agreements, other studies use mean pairwise sensitivities and specificities [3, 4], which can demonstrate non-inferiority of Persyst compared to humans. One study used a reference standard of video-EEG proven epilepsy, ensuring that the IEDs detected by Persyst are true IEDs [5]. These reference standards require opinions from multiple human experts, which is expensive. To minimise costs, most studies use small datasets of preselected, IED-rich EEG records, often from specific settings, such as epilepsy monitoring units. The prevalence of IEDs in these preselected records is not representative of a clinical population, so positive and negative predictive values cannot be calculated [12]. Furthermore, to augment limited data, EEGs are often split into small epochs, which can lead to exaggerated estimates of specificity, as the probability of an individual epoch containing an IED is low.

Routine scalp EEGs have been described as the most useful type of EEG record in the diagnosis of epilepsy [13]. This study, therefore, aims to evaluate the accuracy of Persyst in IED detection in a large dataset of routine, unprocessed, non-preselected EEGs from a representative clinical population. This will allow the calculation of accuracy statistics, including positive and negative predictive values, as well as contributing data on how Persyst performs clinically.

## Methods

### Data extraction

Data were collected retrospectively from EEG archives in the Walton Centre, Liverpool, a tertiary centre for neuroscience in the UK. Permission for accessing and using the data for research was granted locally. EEGs with complete clinical reports that were recorded between June 2024 and February 2025 were used in analysis.

Routine EEG recordings were carried out by clinical physiologists in line with local protocols. EEGs used a 10:20 system modified to include an additional 6 electrodes over the inferior temporal region with a sampling rate of 512 samples per second and bandpass of 0.5-70Hz. Activation techniques (photostimulation and hyperventilation) were used to elicit epileptiform activity, unless patients did not consent, or they were medically contraindicated.

EEG reports included a factual report from the clinical physiologist recording the EEG, which commented on the presence or absence of IEDs. The factual report was subsequently reviewed by a senior clinician, alongside their own interpretation of the EEG, to write a clinical report, which confirmed the presence of IEDs. Senior clinicians were either consultants in neurophysiology, senior registrars training in neurophysiology, clinical scientists, or consultant neurologists with a specialist interest in epilepsy or neurophysiology.

The presence or absence of IEDs according to the factual and clinical reports was recorded. Patient age, gender, EEG indication and other EEG activity were also collected.

### Persyst algorithm

Persyst is a commercially available spike detection software. Raw EEG data were processed by the P15 software. P15 has a proprietary inbuilt artefact reduction system, so manual artefact reduction steps were not required. Persyst has three sensitivity settings for IED detection: low, medium and high. These threshold settings are preset by the manufacturer. Full details on the latest iteration, P15, are proprietary and not published. Details on P13, a previous iteration which is based on a combination of traditional computer code and feed forward neural networks, are available [4].

### Statistical analyses

The neurophysiologist’s factual report and the clinician’s clinical report were used as the reference standards. Cohen’s kappa was used to assess interrater variability between neurophysiologists and senior clinicians. The index tests were the detection of IEDs by Persyst, recorded as a binary outcome, at each sensitivity setting (low, medium and high).

Subsequently, using standard formulae, and using Microsoft Excel, sensitivity, specificity, positive and negative predictive values, and overall accuracy were calculated. Sensitivity and specificity estimates were then used in receiver operating characteristic (ROC) analysis and a value for area under the curve (AUC) was calculated, along with a P-value to test whether AUC was > 0.5. Further subgroup analyses were performed to assess whether mean false positive rates varied according to age, gender, or other EEG abnormalities. The subgroup ‘other EEG abnormalities’ was further subcategorised into ‘focal slowing’, ‘generalised slowing’, ‘all slow activity’ and ‘sharp activity’. Two proportion Z-tests were used to calculate p-values to assess whether differences in mean estimates of false positive rates were statistically significant.

## Results

### Characteristics of included patients

The characteristics of included patients, average EEG length and EEG indication are summarised in Table 2. 234 (48.5%) patients were male, and participants had a median age of 38 (IQR: 32). The majority (95.7%) of patients had the EEG as part of an evaluation for epilepsy.

**Table 2:** Summary of characteristics of included patients, their EEGs and the EEG indication

No. of males (%)	234	(48.45%)		
Median age, years (IQR)	38	(32)		
Median length of EEG recording, minutes (range):	30	(23–62)		
Previous diagnosis of epilepsy (%)	75	(15.50%)		
EEG indication:	Encephalopathy/encephalitis		21	(4.34%)
	Epilepsy:		463	(95.66%)
		Classification	97	(20.04%)
		Diagnostic uncertainty	356	(73.55%)
		Subclinical seizure	5	(1.03%)
		Prognostication	5	(1.03%)

### Characteristics of reported EEGs

Table 3 summarises the findings of the final clinical report of the EEGs. The majority (*n* = 306, 63.22%) were reported as normal, 53 (10.95%) were reported as containing IEDs and 125 (25.83%) were reported as ‘abnormal in another way’. EEGs reported as ‘abnormal in another way’ included EEGs with focal or generalised slow activity, sharpened components and transients associated with sleep. Neurophysiologists reported IEDs in 62 (12.81%) EEG records in the initial factual report. In 9 cases, the senior clinician contradicted the factual report and concluded that IEDs were not present, usually because they identified them to be artefactual, not meeting criteria for IEDs or benign EEG correlates of sleep. There were no cases in which senior clinicians reported IEDs that the neurophysiologist had not identified in the factual report. It should be noted that senior clinicians were not blinded to the factual report when producing the clinical report. Overall, Cohen’s kappa (SE) for interrater agreement between neurophysiologists and senior clinicians was 0.910 (0.0883, *p* < 0.0001).

**Table 3:** Summary of findings of EEG reports

	n	%
Normal	306	63.22%
Reported as containing IEDs	53	10.95%
Abnormal (slow activity, sharpened components, transients associated with sleep etc.)	125	25.83%
Total	484	

### Test accuracy statistics for persyst

Table 4 summarises the test accuracy statistics for Persyst’s performance at detecting IEDs in a routine record, using each of Persyst’s preset sensitivity settings as an index test and the neurophysiologist’s initial factual report as the reference standard.

**Table 4:** Sensitivity, specificity, PPV (positive predictive value), NPV (negative predictive value) and overall accuracy estimates for Persyst's performance at each of its sensitivity settings, using the neurophysiologist’s factual report as reference standard

	Low	(95% CI)	Medium	(95% CI)	High	(95% CI)
Sensitivity	40.3%	(36.0–44.7)	75.8%	(72.0–79.6)	98.4%	(97.3–99.5)
Specificity	86.7%	(83.7–89.8)	60.0%	(55.6–64.3)	29.4%	(25.3–33.4)
PPV	30.9%	(26.8–35.0)	21.8%	(18.1–25.4)	17.0%	(13.7–20.3)
NPV	90.8%	(88.2–93.4)	94.4%	(92.4–96.5)	99.2%	(98.4–100)
Overall accuracy	80.8%	(77.3–84.3)	62.0%	(57.7–66.3)	38.2%	(33.9–42.6)

Similarly, Table 5 summarises estimates of test accuracy statistics but uses the final clinical report as the reference standard. This is what is used clinically to support epilepsy diagnosis and will therefore be used in subsequent analysis.

**Table 5:** Sensitivity, specificity, PPV (positive predictive value), NPV (negative predictive value) and overall accuracy estimates for Persyst's performance at each of its sensitivity settings, using the clinical report as reference standard

	Low	(95% CI)	Medium	(95% CI)	High	(95% CI)
Sensitivity	45.3%	(40.8–49.7)	81.1%	(77.6–84.6)	98.1%	(96.9–99.3)
Specificity	86.8%	(83.8–89.8)	59.9%	(55.5–64.2)	28.8%	(24.7–32.8)
PPV	29.6%	(25.6–33.7)	19.9%	(16.3–23.5)	14.5%	(11.3–17.6)
NPV	92.8%	(90.5–95.1)	96.3%	(94.6–98.0)	99.2%	(98.4–100)
Overall accuracy	82.2%	(78.8–85.6)	62.2%	(57.9–66.5)	36.4%	(57.9–66.5)

### Receiver operating characteristic (ROC) and area under the curve (AUC) analysis

**Fig. 1:**
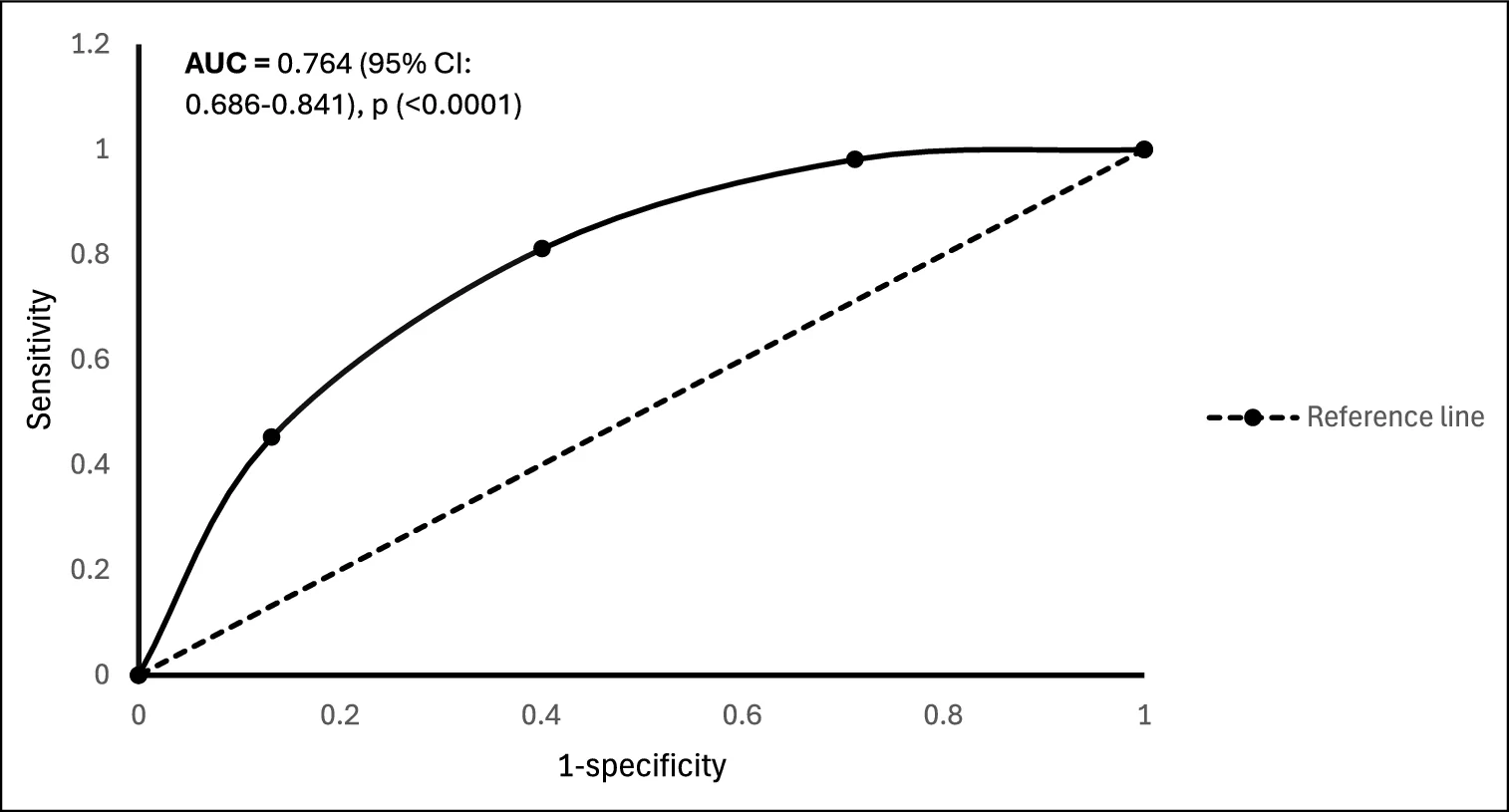
Receiver operating characteristic curve for Persyst's performance at IED detection at each sensitivity setting (low, medium and high), using final clinical report as a reference standard

Figure 1 is a receiver operating characteristic curve showing Persyst’s performance at IED detection using the final clinical report as a reference standard. Area under the curve is 0.764 (95% CI: 0.686–0.841), *p* (< 0.0001).

### Subgroup analyses

To identify factors that may affect Persyst’s performance, subgroup analyses comparing false positive rates in different groups were performed. There was no significant difference in false positive rate between patients with an otherwise normal versus otherwise abnormal EEG (37.6% versus 46.4%, *p* = 0.190), females versus males (39.4% versus 41%, *p* = 0.736), previous epilepsy diagnosis (39.6% versus 43.3%, *p* = 0.586) or age < 50 versus >/= 50 (39.8% vs 40.7%, *p* = 0.843). Further subcategorisation of abnormalities into ‘focal slowing’, ‘generalised slowing’, ‘all slow activity’ and ‘sharp activity’ did not find a significant difference in false positive rate compared to the average false positive rate, but numbers in each group were low.

## Discussion

This study evaluated the utility of a machine learning model approach to IED detection on a large, non-preselected dataset of routine clinically indicated EEGs. At the medium (default) setting and using the final clinical EEG report as the reference standard, Persyst had a sensitivity of 81.1% (95% CI: 77.6–84.6), which is lower than previous comparable studies, which found sensitivity to be between 95–100% [5, 9, 14]. The specificity was 59.9% (95% CI: 55.5–64.2). Specificity in previous comparable studies ranges from 3.33% [5] to 88% [9]. Why the sensitivity and specificity differ from previous evaluations is unclear, but it is likely reflective of varying methodologies, iterations of Persyst, datasets and reference standards.

A key strength of this study is that it evaluates Persyst in a representative clinical population, therefore allowing the accurate calculation of positive and negative predictive values. At Persyst’s default (medium) sensitivity setting, the NPV was 96.3% (95% CI: 94.6–98.0), indicating that Persyst is a good method to rule out IEDs in a routine record. The PPV was 19.9% (95% CI:16.3–23.5). Practically, this means that around four in five IEDs that Persyst identifies are false positives, which could explain low clinician trust in the model [6]. The P15 algorithm is proprietary and IED detection thresholds are preset, meaning it is not possible for a clinician to interrogate how P15 has reached its conclusion, possibly further reducing clinician trust. Furthermore, the use of Persyst to detect IEDs may promote EEG overreading which can lead to the misdiagnosis of epilepsy, which can have many important negative consequences for patients [15].

Based on these findings, box 1 makes suggestions for how Persyst should and should not be used practically.

**Table:**
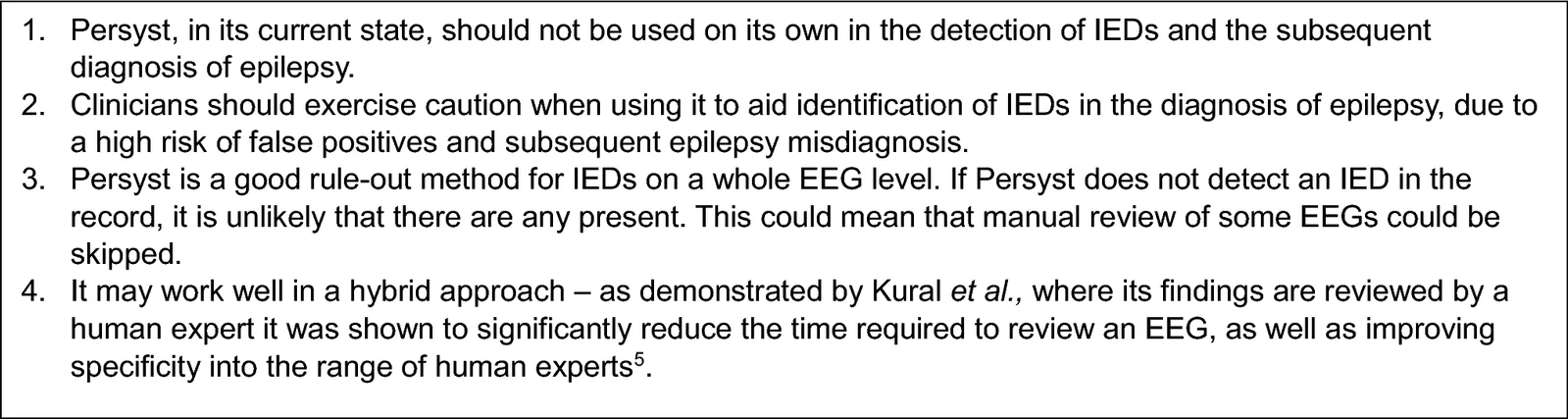
**Box 1: Suggestions, based on this analysis and previous analyses, on how Persyst should and should not be used clinically**

A key limitation of this study was the reference standard, which relied upon conventional clinical reporting from two non-independent reviewers, risking bias. As previously mentioned, there is significant interrater variability in IED detection [3], meaning that this reference standard is likely less robust than consensus agreements and pairwise comparisons used in other studies. Future research could use an external reference standard, but this is resource intensive. The reference standard used in this present study reflects current clinical practice and this study evaluates a greater quantity of EEG records than has been evaluated previously.

A further limitation is the focus on short, routine, outpatient EEGs from a single centre, which may limit the generalisability of the findings. EEGs used in this study had a median length of 30 min and the performance of P15 in longer, ambulatory recordings was not evaluated. Routine, 30 min recordings can already be rapidly reviewed [5], and automated spike detection models will likely have the greatest utility in prolonged, ambulatory EEG recordings. Nevertheless, a prior study did report time saving benefit with the use of P14 in 20-min EEGs [5]*.* It is difficult to generalise the findings from this study of routine EEGs to prolonged ambulatory recordings, but it is likely that the number of falsely detected IEDs will rise due to increasing interference and artefact. Future studies should therefore evaluate the performance of spike detection models in prolonged recordings.

Presently, Persyst produces too many false positives to be used in isolation. A hybrid approach, using Persyst supervised by a clinician, has previously been demonstrated to be non-inferior to full conventional review, with a significant time-saving benefit for IED detection, but this was in a preselected dataset of EEGs [5]. The high false positive rate of P15 observed in this present study could paradoxically increase a neurophysiologist’s time to review, as well as promote EEG overreading and subsequent epilepsy misdiagnosis. Additionally, although it has previously been demonstrated that Persyst can save time in IED detection[5], it is unclear whether Persyst saves time overall, as a neurophysiologist must still report other features of the EEG. Data on the time to review were not recorded in this study. Future research should therefore evaluate a hybrid approach in a clinical population and whether this leads to a subsequent optimisation of clinical workflows.

Finally, this study provides useful estimates of test accuracy statistics of P15 in a routine, clinical dataset. However, the use of a non-preselected dataset with low rates of true IEDs, led to wide confidence intervals. Future evaluations should use larger testing datasets to increase the precision of these estimates.

## Conclusion

This evaluation of Persyst’s accuracy provides useful data on its performance in a large dataset of routine EEGs. The high NPV suggests that Persyst is a good rule-out method for IEDs in a routine record, potentially removing the need for human review. The low PPV found in this study helps explain a lack of clinician trust in Persyst. Future work should focus on evaluating a human-Persyst hybrid approach to IED detection, particularly to ensure that a high number of false detections produced by Persyst does not increase rates of epilepsy misdiagnosis. Ideally, this evaluation should be done in a large dataset of prolonged EEGs, where automated IED detection is likely to be of greatest use clinically and misclassification of artefacts remains a key challenge. Data on the time to review should also be collected. This approach will allow a more comprehensive evaluation of the safety and efficacy of introducing automated spike detection models into clinical workflows.
